# CT-based delta-radiomics nomogram to predict pathological complete response after neoadjuvant chemoradiotherapy in esophageal squamous cell carcinoma patients

**DOI:** 10.1186/s12967-024-05392-4

**Published:** 2024-06-18

**Authors:** Liyuan Fan, Zhe Yang, Minghui Chang, Zheng Chen, Qiang Wen

**Affiliations:** 1 Department of Thoracic Surgery, Qilu Hospital of Shandong University, Jinan, 250012 Shandong China; 2 Department of Radiation Oncology, Shandong Provincial Hospital Affiliated to Shandong First Medical University, 324 Jingwu Road, Jinan, 250021 Shandong China

**Keywords:** Delta-radiomics, Neoadjuvant chemoradiotherapy, Esophageal squamous cell carcinoma, Pathological complete response, Computed tomography

## Abstract

**Background:**

This study developed a nomogram model using CT-based delta-radiomics features and clinical factors to predict pathological complete response (pCR) in esophageal squamous cell carcinoma (ESCC) patients receiving neoadjuvant chemoradiotherapy (nCRT).

**Methods:**

The study retrospectively analyzed 232 ESCC patients who underwent pretreatment and post-treatment CT scans. Patients were divided into training (n = 186) and validation (n = 46) sets through fivefold cross-validation. 837 radiomics features were extracted from regions of interest (ROIs) delineations on CT images before and after nCRT to calculate delta values. The LASSO algorithm selected delta-radiomics features (DRF) based on classification performance. Logistic regression constructed a nomogram incorporating DRFs and clinical factors. Receiver operating characteristic (ROC) and area under the curve (AUC) analyses evaluated nomogram performance for predicting pCR.

**Results:**

No significant differences existed between the training and validation datasets. The 4-feature delta-radiomics signature (DRS) demonstrated good predictive accuracy for pCR, with α-binormal-based and empirical AUCs of 0.871 and 0.869. T-stage (*p* = 0.001) and differentiation degree (*p* = 0.018) were independent predictors of pCR. The nomogram combined the DRS and clinical factors improved the classification performance in the training dataset (AUC_αbin_ = 0.933 and AUC_emp_ = 0.941). The validation set showed similar performance with AUCs of 0.958 and 0.962.

**Conclusions:**

The CT-based delta-radiomics nomogram model with clinical factors provided high predictive accuracy for pCR in ESCC patients after nCRT.

## Background

Esophageal cancer (EC) ranks is the seventh most common cancer in terms of incidence and the sixth leading cause of death worldwide, with approximately 604,000 new cases and 544,000 deaths each year [1]. Particularly in China, around 478,000 new esophageal squamous cell carcinoma (ESCC) diagnoses occur annually [2]. Squamous cell carcinoma (SCC) accounts for roughly 90% of all esophageal cancer cases globally [3]. Despite advances in screening, treatment modalities like chemoradiotherapy and immunotherapy, 5-year survival remains under 20% for locally advanced disease due to tumor heterogeneity and treatment resistance [4]. Therefore, exploring novel advanced treatment modalities, effectively predicting therapeutic efficacy, and precisely stratifying patient are imperative to improve prognosis in locally advanced ESCC.

Neoadjuvant chemoradiotherapy (nCRT) followed by esophagectomy is the standard treatment strategy for patients with resectable locally advanced ESCC (T1, N1–3, M0; or T2–4a, N0–3, M0) [5]. Compared to surgery alone, nCRT can increase locoregional control rates and prolong overall survival through tumor downsizing and downstaging [6]. Pathological complete response (pCR) is a crucial indicator of nCRT efficacy. Patients achieving pCR have lower recurrence rates and longer survival than those with partial response and non-response [7]. Moreover, studies suggest comparable outcomes for wait-and-see versus surgery in pCR patients, highlighting pCR's vital role in guiding treatment decisions. However, due to tumor heterogeneity and individual differences, only 25 to 40% of cases achieve pCR [8]. Patients who have no response to neoadjuvant chemoradiotherapy have worse prognosis than surgery alone [9]. Hence, it is imperative to predict treatment response to nCRT and discern which patients with ESCC may attain a pCR.

Although various methods are currently available to assess response to nCRT in ESCC, several limitations exist that cannot be ignored. Firstly, traditional endoscopic ultrasound (EUS) and endoscopy may have reduced accuracy and objectivity in reflecting true treatment sensitivity, as they can be confounded by inflammation, edema, and fibrosis related to nCRT treatment effects [10]. Secondly, invasive examinations such as endoscopy and biopsy can be challenging to successfully perform in patients with nCRT-induced esophagitis or luminal stenosis [11]. Imaging modalities such as computed tomography (CT) have been used to measure tumor diameter and volume changes after nCRT, but these parameters have resulted in unsatisfactory sensitivity and specificity. Therefore, clinical treatment decisions for ESCC patients following nCRT cannot be based solely on these existing methods, and more accurate predictive tools are needed.

Artificial intelligence (AI) has been successfully applied in medicine [12], enabling automated recognition and processing of complex medical data such as images, genetics, and metabolism [13]. Machine learning (ML), a critical artificial intelligence branch, uses computational and mathematical models to analyze multi-scale data through self-learning, achieving classification and prediction. Radiomics combines artificial intelligence and medical imaging to extract quantitative image features using algorithms, potentially reflecting underlying pathophysiology and revealing pathogenesis [14]. The development of CT radiomics has provided a new scope for tumor-related differential diagnosis, prognosis prediction and exploring gene expression [15–17]. Combining ML algorithms and CT is a promising tool to improve prediction in esophageal cancer. Recent radiomics models used single-phase pretreatment scans to predict chemoradiotherapy response, ignoring the alternations of tumors during treatment or follow-up [18–20].

Comparing radiomics features from pre- and post-treatment scans provides information on microenvironment changes and may better predict response. This radiomics subfield has been identified to be predictive of treatment response in many types of cancer, including rectal adenocarcinoma [21] and gastric cancer [22]. To the best of our knowledge, no prior studies have explored the potential of CT delta-radiomics features in predicting pCR for ESCC patients subjected to nCRT. Accurately identifying nCRT responders could maximize benefits and avoid unnecessary toxicities for non-responders.

Thus, this retrospective study aimed to develop and validate a nomogram as a non-invasive tool combining clinical information and delta-radiomics features from baseline and post-nCRT CT scans to predict pCR in locally advanced ESCC. By enabling personalized prediction of nCRT sensitivity, this nomogram can facilitate selecting optimal candidates. The nomogram has potential to significantly improve risk stratification, guide individualized treatment decisions, and ultimately improve survival outcomes for ESCC patients.

## Methods

### Patient selection

This study retrospectively analyzed 304 patients with locally advanced ESCC who underwent nCRT followed by surgery between June 2018 and December 2021. Inclusion criteria were as follows: (1) pathological diagnosis by endoscopic biopsy; (2) clinical staging by EUS and CT from neck to abdomen; (3) no distant metastasis confirmed by whole-body positron emission tomography and computed tomography (PET/CT) or cranial magnetic resonance imaging (MRI); (4) neck ultrasonography with lymph node fine needle aspiration if indicated; (5) measurable lesion > 1 cm on CT; (6) all patients received standard nCRT followed by esophagectomy. Additionally, we excluded a portion of patients based on the following criteria: 21 patients had a history of disease-related treatment; 7 patients had low-quality CT images; 11 patients couldn't accurately delineate tumor borders; 24 patients did not complete the standard nCRT treatment; and 9 patients had an interval between post-treatment CT and surgery longer than 6 weeks. Therefore, the final analysis included a total of 232 patients in the study cohort. Table 1 furnishes comprehensive details regarding recorded clinical factors, including gender, location, and T staging.

**Table 1:** Clinical factors of patients with non-pCR and pCR in the training and validation datasets

Characteristic	Training dataset	Validation dataset
Gender		
Male	139	37
Female	47	9
ECOG PS		
0–1	144	38
2	42	8
Alcohol history		
Yes	114	34
No	72	12
T stage		
T1-2	43	5
T3	81	19
T4	62	22
N staging		
0–1	66	20
2–3	120	26
Degree of differentiation		
Low	47	13
Middle-high	139	33
Location		
Upper	26	14
Middle	58	16
Lower	102	16
nCRT response		
Non-pCR pCR	127 59	29 17

*ECOG PS* Eastern Cooperative Oncology Group Performance Status, *nCRT* neoadjuvant chemoradiotherapy, *pCR* pathological complete response

### Radiotherapy delivery and chemotherapy administration

All patients with TNM staging of T1, N1–3, M0 or T2–4a, N0–3, M0 were treated with nCRT. Radiotherapy plans were generated using the Varian treatment planning system (Varian Medical System, Inc., Palo Alto, California, USA) The target volume included the gross tumor volume and metastatic lymph nodes. A total radiation dose of 45 Gy was delivered by intensity-modulated radiotherapy (IMRT) with 6–15 MV X-rays over 5 weeks, with 1.8 Gy per fraction and 5 fractions administered per week.

All patients received concurrent chemotherapy with radiotherapy, consisting of 5 cycles of intravenous chemotherapy before response assessment. The chemotherapy regimen included paclitaxel at a dose of 50 mg/m^2^ and carboplatin dosed based on an area under the curve (AUC) of 2 mg/ml/min, administered on days 1, 8, 15, 22 and 29. Chemotherapy doses and strategies were adjusted based on individual toxicity levels and blood counts.

Radical esophagectomy was typically scheduled 6 to 8 weeks after completion of nCRT, allowing sufficient time for patients to recover before undergoing the surgical procedure. The surgical procedure involved either an open or minimally invasive transthoracic esophagectomy, accompanied by a two-field lymph node dissection. To improve local control and enhance prognosis, at least 15 lymph nodes were removed during the surgery.

### Pathological evaluation and response assessment

Resected tissues were subjected to pathological evaluation by two independent pathologists with 8 and 12 years of experience who were blinded to the clinical data. Pathological complete response (pCR) indicates no viable cancer cells at the primary site and lymph nodes. Preoperative chemoradiotherapy response was assessed using the 5-point Mandard tumor regression grade (TRG) scale [23]: Grade 1 indicated a complete pathological response with no residual tumor cells; Grade 2 represented the presence of very few tumor cells scattered in fibrosis; Grade 3 meant a larger amount of residual tumor cells, but comprising a smaller proportion than fibrosis; Grade 4 illustrated a higher proportion of residual tumor cells compared to fibrosis; Grade 5 was almost all tumor cells with little or no fibrosis present. pCR was defined as TRG Grade 1 with negative lymph nodes.

### CT image acquisition and regions of interest segmentation

All patients underwent pre- and post-treatment CT. Pre-treatment CT was performed 1–2 weeks before starting nCRT. To minimize effects of radiation-induced inflammation, post-treatment CT was done 4–6 weeks after completing nCRT. The CT images were obtained using a 128-row CT scanner in the axial, coronal and sagittal planes (Philips iCT 128, Philips Medical System, The Netherlands) with 120 kV voltage, 300–400 mA current, 3 mm slice thickness, and 512 × 512 matrix.

Regions of interest (ROIs) encompassed the entire primary tumor volume, defined slice by slice on lung (1500/− 500 Hu) and mediastinal (300/− 60 Hu) window settings. Pre- and post-treatment ROIs were automatically contoured using AccuContour software (version 3.0, Manteia Medical Technologies Co. Ltd., Xiamen, China) on the basis of deep learning algorithms, then manually modified by two radiologists with 10 and 15 years of experience. Two independent radiologists were blinded to the clinical data and pathological information, and any discrepancy ≥ 5% was resolved by consensus [24, 25].

### Radiomics features extraction

CT radiomics feature extraction was automatically performed with PyRadiomics packages, which enable feature calculation in 3D Slicer software (Version 4.10, http://www.slicer.org). A total of 93 features were extracted from each ROI, including 18 first-order intensity histogram (IH) and statistical matrix (SM) features, 24 grey-level co-occurrence matrix (GLCM), 16 Gy-level run-length matrix (GLRLM), 16 Gy-level size zone matrix (GLSZM), 5 neighboring gray-tone difference matrix (NGTDM), and 14 Gy-level dependence matrix (GLDM) features. Additionally, 744 wavelet features for IH and SM were extracted from 8 wavelet decompositions. All features were z-score normalized to a mean of 0 and standard deviation of 1. To assess intra-observer reproducibility, two radiologists independently performed ROIs delineation. Both radiologists were blind to the clinical and histopathological data. They each segmented CT images of esophageal cancer in 30 randomly selected samples. Radiomics features from the two ROIs were compared using intra-class correlation coefficients (ICCs). Features with ICC ≥ 0.8 were retained as having almost perfect agreement. Features with ICC < 0.8 were initially eliminated before further analysis.

The changes in the radiomic features (delta-radiomics features, DRF) were calculated from the differences between posttreatment CT (Post-nCRT radiomics features, RF_*post*_) and pre-treatment CT (Pre-nCRT radiomics features, RF_*pre*_):

1 $${RF}_{post}-{RF}_{pre}=DRF$$

### Delta-radiomics features selection

To maintain an effective and robust delta-radiomics signature (DRS), feature selection was employed to identify and eliminate irrelevant features that could reduce performance for identifying pathological complete response (pCR). Ideal features were first selected based on univariate logistic regression between pCR and non-pCR patients in the training dataset, using a threshold of 0.1 to avoid removing highly discriminative features before multivariable analysis. To reduce overfitting and selection bias, the least absolute shrinkage and selection operator (LASSO) machine learning method was then utilized to select optimal features capable of distinguishing between pCR and non-pCR [26]. For the binary logistic regression, the tuning parameter λ was chosen in LASSO through fivefold cross-validation based on minimum criteria using the “glmnet” package in R software (Version 3.4, http://www.r-project.org/) [27].

### Delta-radiomics signature and nomogram construction

A logistic regression model was constructed and evaluated for the predictive performance of the selected radiomics features constituting the DRS. The DRS was calculated for each patient as the linear combination of features weighted by corresponding LASSO coefficients.

To determine if adding clinical factors could improve differentiating pathological response beyond the DRS alone, a nomogram was built combining DRS and clinically significant factors identified through multivariable analysis. Then, a nomogram was established in accordance with multivariable analysis to predict the probability of pCR.

### Delta-radiomics signature and nomogram validation

The neoadjuvant chemoradiotherapy (nCRT) response prediction performance was evaluated in the validation dataset using receiver operating characteristic (ROC) curve analysis. Given the limited sample size, bias from uneven distribution between groups was inevitable, potentially underestimating or overestimating performance. When the positive and negative data used to construct the classifying model were not balanced, the ROC curves may manifest poorly in the evaluation with a high AUC value. In this situation, it was recommended to plot positive predictive value of all thresholds against true positive using a precision-recall curve (PRC), and the area under the PRC was named the average precision (AP). In the present study, we plotted the smooth ROC curve and PRC on the basis of α-binormal model, which solved the above problems. The discrimination of the nomogram was evaluated with the α-binormal model-based ROC curve and PRC. The nomogram was obtained from the training dataset and then tested in the validation dataset, and the total points for every patient were calculated. Agreement between predicted and actual pCR probability was assessed by calibration curves through restricted cubic splines. In addition to the calibration curve, we reported a prediction metric AUC that reflects the calibration of the model.

### Statistical analysis

Statistical analysis was performed using SPSS v21.0 (Chicago, USA) and R software v3.4 (Auckland, New Zealand, http://www.r-project.org/). Categorical variables were compared between groups using chi-squared or Fisher’s exact tests. Continuous variables were compared using Mann–Whitney U tests. Comparison, calibration and precision-recall curves were generated with the “rms” and “pROC” packages. All the P-values were two-sided, and the results were considered statistically significant when the *p*-values were less than 0.05.

## Results

### Patient characteristics

This study included 232 patients, with 156 non-pCR and 76 pCR cases. The patients were divided into a training dataset consisting of 186 patients and a validation dataset consisting of 46 patients. The cohort included 176 males and 56 females. Using TNM staging, 48 patients were classified as T1-2, while the numbers of T3 and T4 were similar at 100 and 84, respectively. Most patients (n = 172) had moderate or well-differentiated tumors, with few poorly differentiated cases. Table 1 lists the clinical variables, which did not significantly differ between the training and validation sets (*p* > 0.05), including differences in gender, location and other factors.

### Radiomics signature construction and validation

**Fig. 1:**
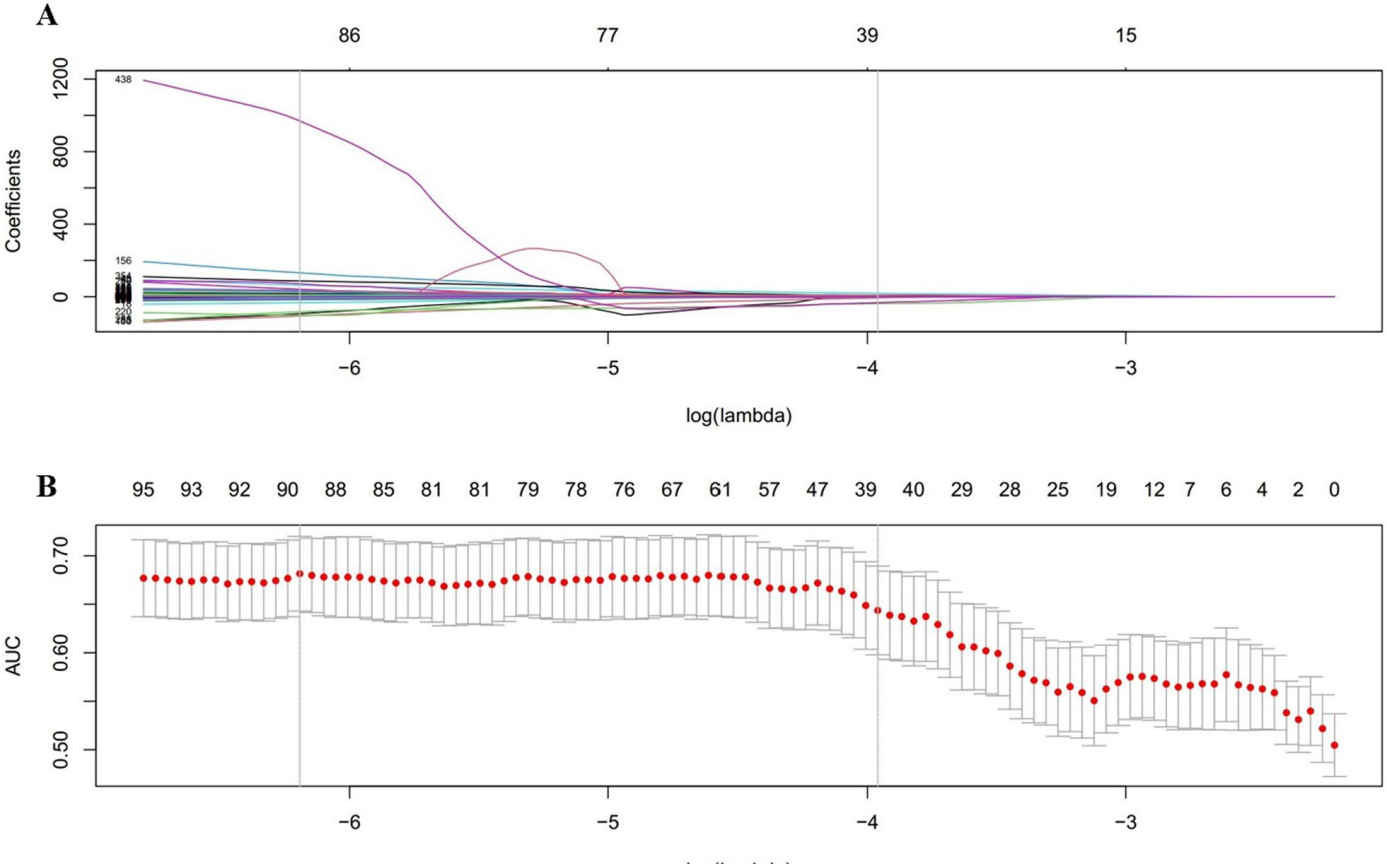
Features selection through LASSO with a binary regression model. **A** The LASSO coefficient profile plot was produced against the log lambda sequence. **B** Tuning parameter (log lambda) selection in the LASSO via minimum criteria. *AUC* area under the curve

Four delta-radiomics features with non-zero LASSO regression coefficients were selected to construct the signature in the training set. Features were weighted by their coefficients, shown in Fig. 1.

**Fig. 2:**
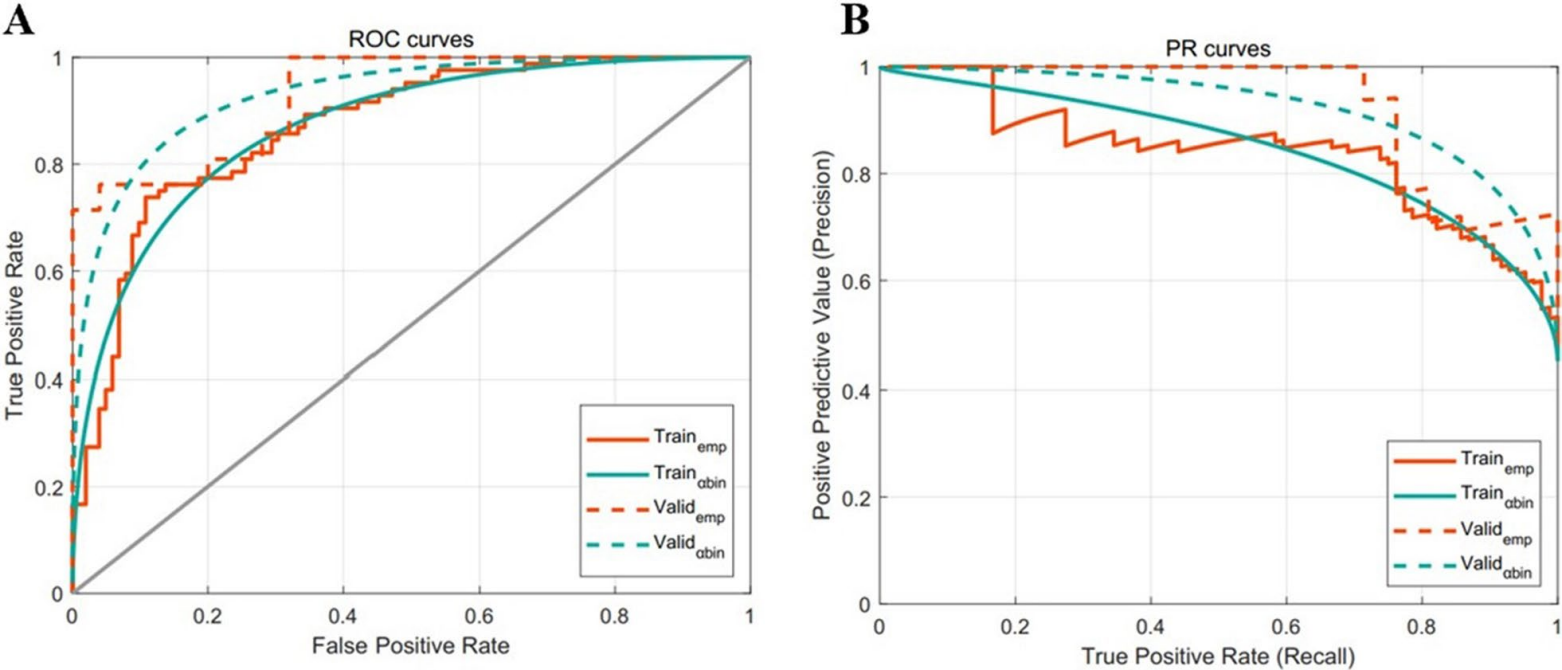
The performances of the developed delta-radiomics signature. **A** Receiver operating characteristics (ROC) curves. **B** Precision-recall curve (PRC)

Figure 2 displays α-binormal-based and empirical ROC and PRC for the delta-radiomics signature. The α-binormal and empirical AUCs and APs of the delta-Rad score are summarized in Table 2. The delta-Rad score exhibited a high efficiency for differentiating pCR and non-pCR according to AUC_αbin_ = 0.871 and AUC_emp_ = 0.869. This performance was confirmed in the validation set, and the aforementioned radiomics yielded good results with AUC_αbin_ = 0.911 and AUC_emp_ = 0.929. Overall, the delta-radiomics signature exhibited favorable diagnostic performance for distinguishing between non-pCR and pCR.

**Table 2:** Comparison of the performances of delta-radiomics signature and nomogram

Performance	Training dataset				Validation dataset			
	AUCα-bin	AUCemp	APα-bin	APemp	AUCα-bin	AUCemp	APα-bin	APemp
DRS	0.871	0.869	0.848	0.826	0.911	0.929	0.921	0.883
Nomogram	0.933	0.941	0.930	0.918	0.958	0.962	0.953	0.914
p-value	< 0.001	< 0.001	< 0.001	< 0.001	< 0.001	< 0.001	< 0.001	< 0.001

*DRS* Delta-radiomics signature, *AUC* Area under the curve, *AP* Average precision, *AUCα-bin* The α-binormal area under the curve, *AUCemp* The empirical area under the curve, *APα-bin* The α-binormal average precision, *APemp* The empirical average precision

### Nomogram development and validation

The results of the relationship between clinical factors and treatment response types in the training dataset are shown in Table 3. Chi-squared and Mann–Whitney U tests were used for univariate analysis to establish the relationship between clinical factors and therapeutic response. No significant difference was found between non-pCR and pCR groups by gender (*p* = 0.365) or ECOG PS (*p* = 0.189). However, T staging showed marked differences between non-pCR and pCR groups (*p* = 0.001) with most non-pCR cases distributed in T3 and T4, while pCR patients were predominantly T1. Notably, the degree of differentiation was higher in pCR patients compared to non-pCR with *p* value = 0.018. No significant difference in N staging was seen between groups (*p* = 0.192), and location was not a predictive factor (*p* = 0.483).

**Table 3:** Clinical factors of patients with non-pCR and pCR in the training dataset

Characteristic	Non-pCR	pCR	*p*
Gender			0.365
Male	92	47	
Female	35	12	
ECOG PS			0.189
0–1	102	42	
2	25	17	
Alcohol history			0.520
Yes	80	34	
No	47	25	
T stage			0.001
T1-2	19	24	
T3	61	20	
T4	47	15	
N staging			0.192
0–1	41	25	
2–3	86	34	
Degree of differentiation			0.018
Low	39	8	
Middle-high	88	51	
Location			0.483
Upper	18	8	
Middle	36	22	
Lower	73	29	

*ECOG PS* Eastern Cooperative Oncology Group Performance Status, *nCRT* neoadjuvant chemoradiotherapy, *pCR* pathological complete response

**Fig. 3:**
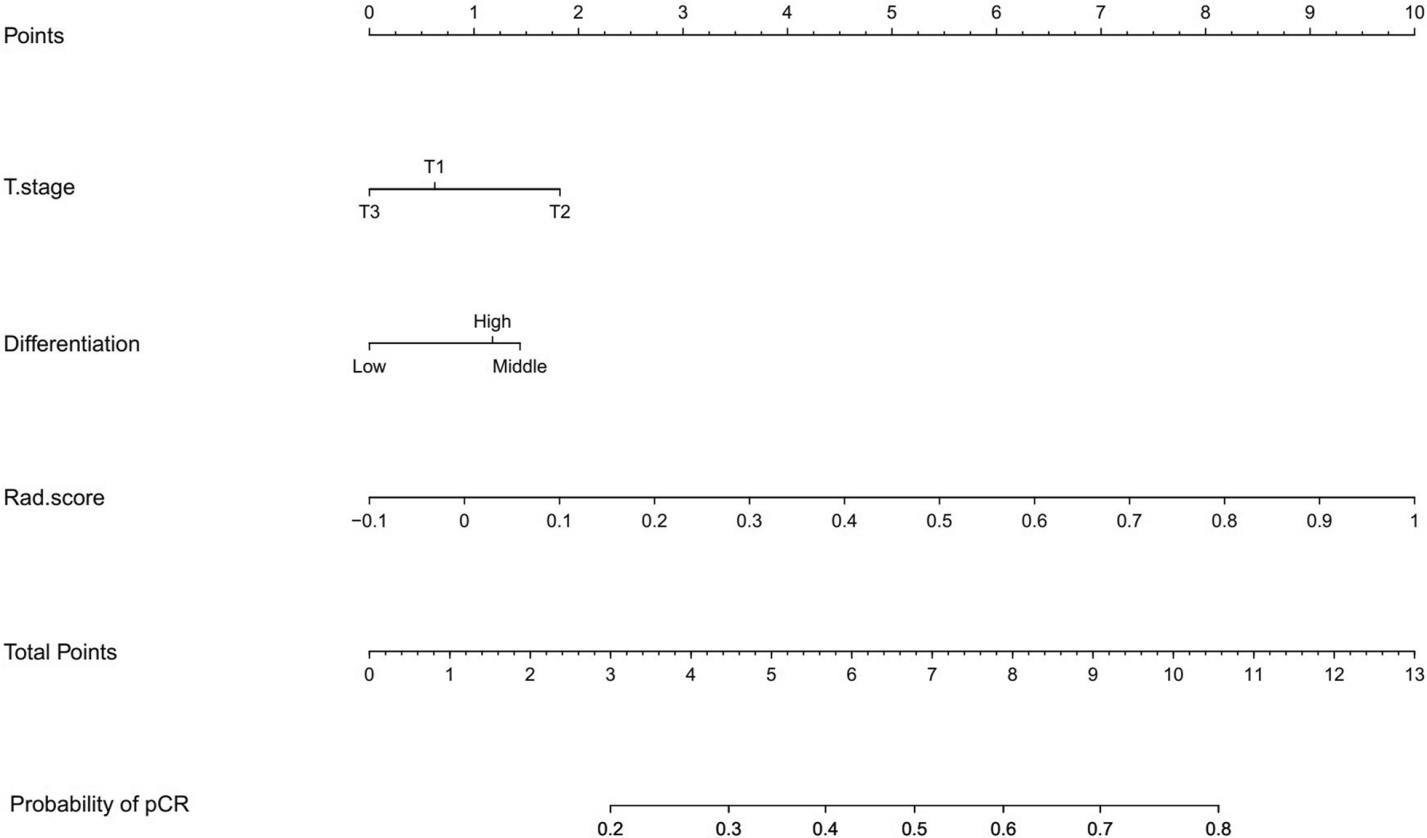
Nomograms developed in this study using the training dataset

**Fig. 4:**
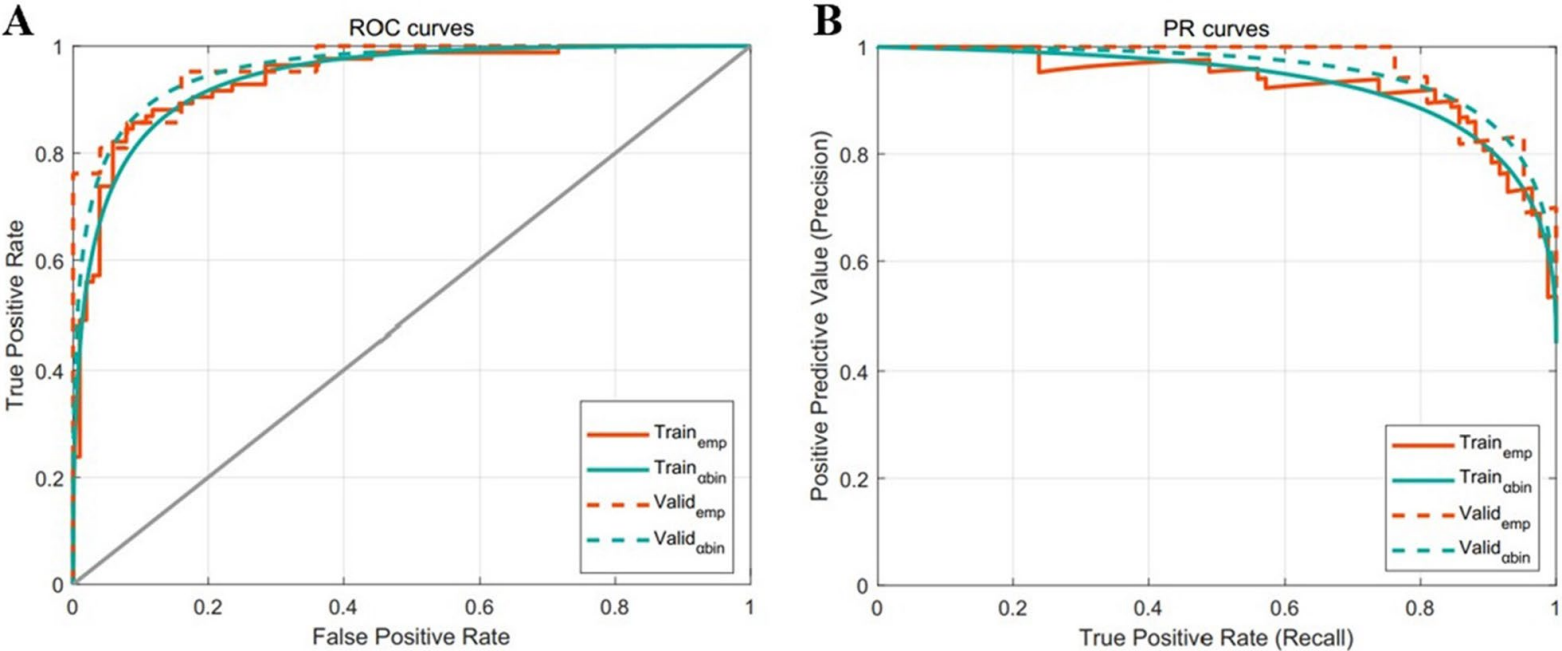
The performance of the developed nomogram. **A** Receiver operating characteristics (ROC) curves. **B** Precision-recall curve (PRC)

The delta-radiomics signatures and independent clinical factors were combined to create a nomogram (Fig. 3). In training samples, the α-binormal and empirical AUCs of the nomogram were 0.933 and 0.941, substantially higher than the DRS alone. The nomogram was also validated in an external cohort, demonstrating superior discriminative ability with AUC_αbin_ = 0.958 and AUC_emp_ = 0.962. As shown in Fig. 4, a clear distinction between non-pCR and pCR was seen in the validation cohort with APs of 0.953 and 0.914, respectively.

**Fig. 5:**
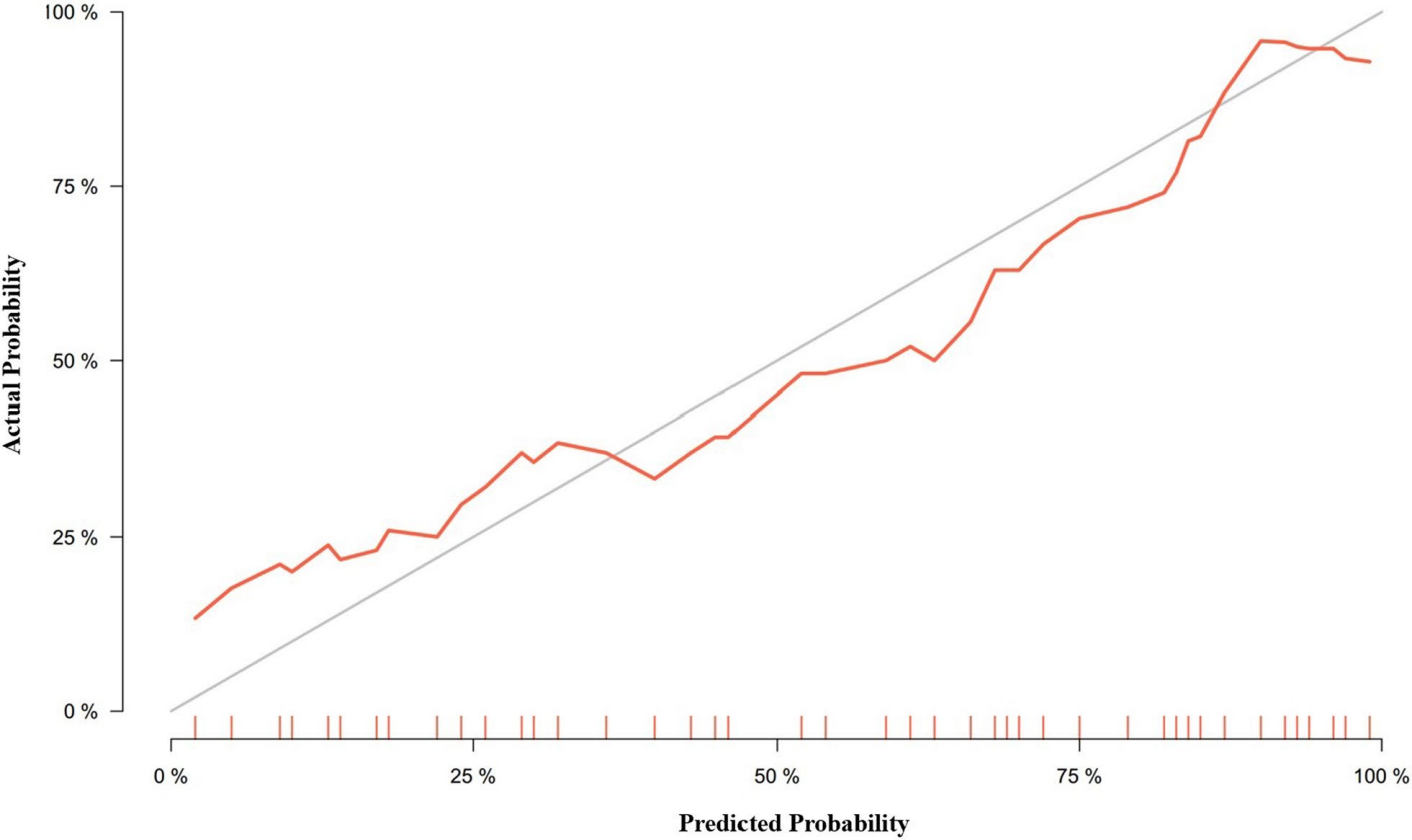
Calibration curve of the nomogram shows as a red line

**Fig. 6:**
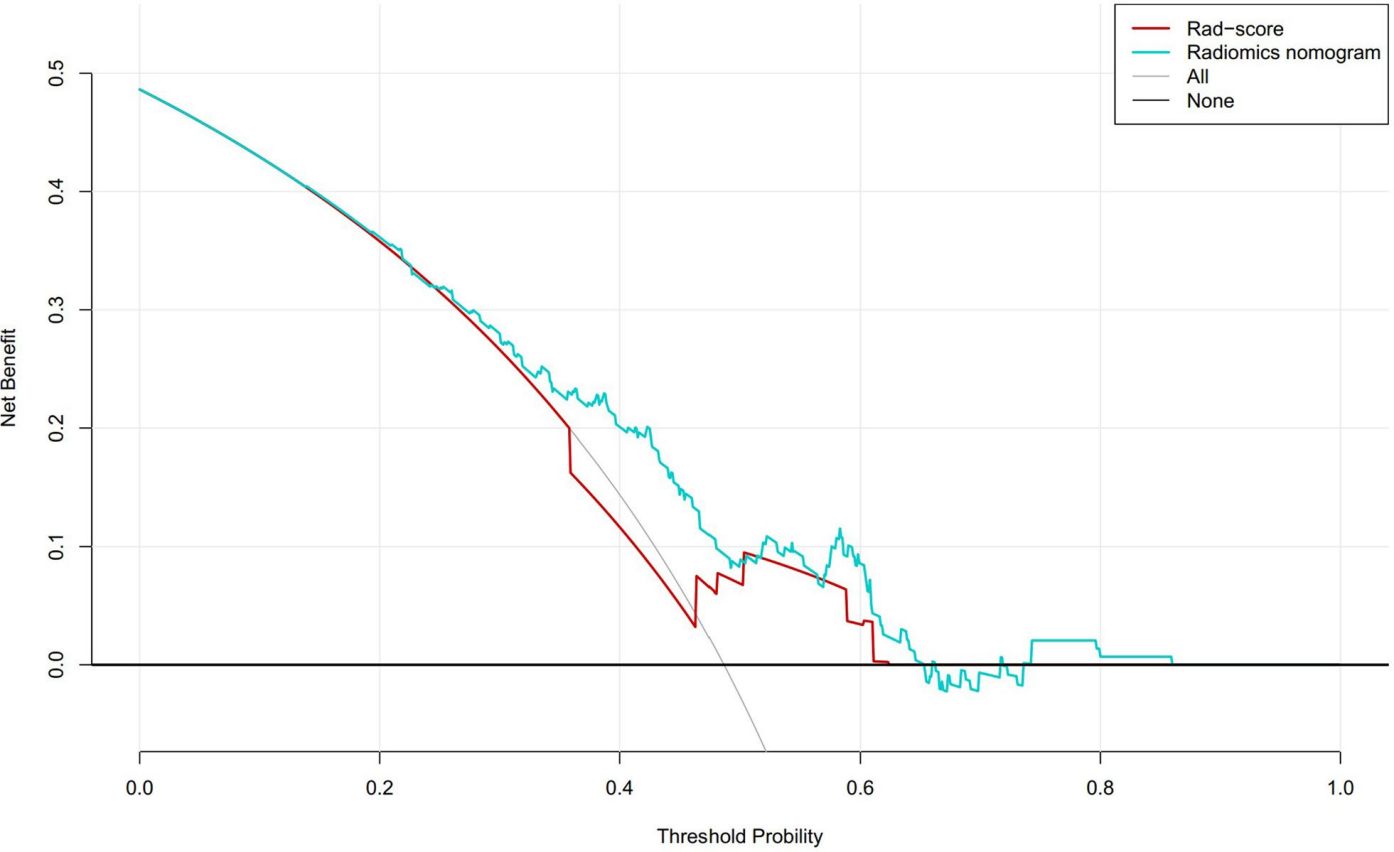
The decision of the delta-radiomics signature, nomogram and two extreme curves were plotted based on the validation dataset. The figure illustrated that the utilize of nomogram to predict pCR probability has a greater benefit that the delta-radiomics signature

The calibration curve showed good agreement between estimated pCR probability and actual observed results by restricted cubic splines, AUC = 0.870 (95% CI: 0.781–0.959) (Fig. 5). The distance of the calibration curve from the diagonal was inversely correlated to the predictive ability of the nomogram. Decision curves were plotted for both the DRS and the nomogram to assess their clinical utility. The results above confirmed that the nomogram exhibited optimal performance in discriminating non-pCR from pCR. yielding a greater net benefit compared to the DRS (Fig. 6).

## Discussion

Early prediction of treatment response in locally advanced esophageal cancer to nCRT is crucial for adjusting treatment strategies. Patients achieving pCR may avoid esophagectomy and preserve the esophagus. This study aimed to develop and validate a CT-based delta-radiomics score to effectively predict pCR in ESCC patients. Additionally, combining it with clinical variables improved predictive performance compared to the DRS alone, as evidenced in independent validation sets. Therefore, the nomogram incorporating clinical and delta-radiomics features may provide an effective, non-invasive tool to guide clinical decision-making. Adoption of this nomogram into routine clinical practice could significantly enhance current standards of patient care by enabling personalized therapy. This has the potential to improve clinical response rates, optimize health outcomes, prolong survival, and preserve quality of life for patients. The predictive model therefore constitutes a major step forward towards precision oncology and individualized treatment in this deadly disease.

Previous studies assessed CT and EUS for evaluating ESCC response to nCRT based on tumor volume and diameter reduction [28, 29]. However, visual assessment can be influenced by inter- and intra-observer variability, and volume reduction does not always correlate with response. Furthermore, PET/CT and diffusion-weighted imaging magnetic resonance imaging (DWI-MRI) displayed a good performance in predicting pathological response to nCRT [30], but the expensive medical examination fees limit accessibility and affordability. Besides that, perfusion CT and dual energy CT (DECT) were also applied to predict the response of ESCC after nCRT. However, there are some important limitations for CT perfusion. In particular, results may vary depending on the proprietary software used, making comparisons between studies difficult. There are also issues with reproducibility of CT perfusion techniques that need to be addressed [31]. On the other hand, DECT is limited since not all tumor lesions exhibit hypervascularity, and vascularity reduction after chemoradiotherapy does not always strictly relate to response [32, 33].

As a novel imaging quantification method, previous studies have illustrated the potential value of radiomics in predicting histological response to nCRT in esophageal cancer. Hu et al. used CT-based radiomics features and constructed a model to differentiate between ESCC patients with non-pCR and those with pCR, achieving an AUC of 0.852. However, they did not involve any clinical parameters in the model and the efficacy of the models was not explicitly confirmed through external validation [34]. Luo et al. extracted 851 radiomics features from pretreatment CT to build a model for predicting the response of esophageal cancer to nCRT. The model contained 7 radiomics features and had an AUC score of 0.844. But the sample sizes of these studies were relatively small, which increased the risk of model overfitting [35]. A research by van Rossum et al. reported a predictive model of tumor response via ^18^F-FDG PET radiomics features of patients with esophageal cancer, and a corrected c-index of 0.77 was achieved. However, adenocarcinoma accounted for most cases in this study, which has significantly different tumor biology and response to neoadjuvant chemoradiotherapy compared to esophageal squamous cell carcinoma (pCR rate of 27% versus 43.2%, respectively) [36]. Therefore, selecting only one histological type of esophageal cancer for analysis may improve the performance of the model in predicting pCR.

The results of our study indicate that the model based on delta radiomics features had higher predictive power than previous studies, especially when combined with clinical factors. DRF can provide information on heterogeneous changes, which is ignored by single time-point models. Nardone et al. proposed that delta radiomics based on CT improved accuracy for pCR prediction, reporting AUCs of 0.87 and 0.88 in the training and validation sets, respectively [37]. Guo et al*.* performed a study predicting pCR using delta-radiomics features extracted from pre-treatment and post-treatment DCE-MRI in breast cancer patients undergoing neoadjuvant treatment, achieving highest AUCs of 0.917 and 0.842 for the training and validation sets [38]. Moreover, Shen et al. developed a model on an internal cohort of 132 advanced gastric cancer patients and validated it on 45 external patients. The model incorporated CT-based delta radiomics and clinical factors to predict overall survival, with AUC values of 0.827 and 0.853 for internal and external datasets [39]. To our knowledge, no previous studies have assessed delta-radiomics features of CT images for evaluating tumor response in ESCC patients undergoing nCRT. Calculating feature differences before and after treatment using DRF may provide more detailed information on treatment response compared to static radiomics analysis.

Tumor staging is widely known as the most important prognostic indicator for patients with malignant tumors, and it serves as the foundation for clinicians to develop treatment strategies. Previous studies showed patients with earlier T stages before treatment have a higher chance of achieving pCR after chemoradiotherapy [40, 41]. Initial findings by Szumilo et al. revealed tumor invasion depth as the only clinical variable significantly correlated with neoadjuvant chemotherapy response in thoracic ESCC [42]. American joint committee on cancer (AJCC) T staging depends on tumor infiltration depth, considering only the horizontal axis, rather than vertical axis [43]. Consequently, it may be difficult to fully evaluate esophageal cancer due to the lack of partial prognostic information. Radiomics analysis can extract high-throughput and quantitative features of malignancy lesions, reflecting tumor information and improving predictive efficacy. Our results state that T-stage has a close relationship with pCR (*p* = 0.014), and the combination of radiomics and clinical parameters can be applied to further improve predictive performance, surpassing the performance of independent models with an AUC of 0.963. More advanced T stages are associated with larger tumor volumes, higher heterogeneity, poorer differentiation, and increased radiotherapy resistance, reducing radiosensitivity. This conclusion is generally consistent with the study by Luo et al*.* [35]. In the present study, our results revealed higher differentiation degree could serve as a biomarker of nCRT response with *p* = 0.041. Different differentiation statuses influence apoptotic pathways, causing varying treatment responses. Poorly differentiated tumor may exhibit increased expression of DNA repair enzymes and proteins conferring radiotherapy resistance. This enhanced expression enables them to more efficiently repair DNA damage induced by radiotherapy [44]. The superior performance of the combined model may relate to tumor heterogeneity, reflected by radiomics and clinical features describing biological characteristics like cell cycle and chemokine signaling [45].

In addition to T stage and tumor differentiation we mentioned, other clinical factors may associate with neoadjuvant chemoradiotherapy response in esophageal cancer. Although ECOG performance status is an established prognostic factor in cancer, its utility for predicting outcomes in ESCC patients undergoing nCRT is unclear.. Poorer ECOG PS often indicates worse chemoradiation outcomes, as it associates with increased sensitivity to treatment toxicities and complications [46]. Patients with good ECOG PS can better tolerate full-dose radiotherapy and complete chemotherapy cycles [47]. However, there are certain tumor types, such as small cell lung cancer, that exhibit high responsiveness to chemoradiotherapy, thus presenting potential exceptions [48]. Given the different mechanisms of etoposide and paclitaxel, tolerability within ECOG cohorts may depend on the regimen specifics. More research is needed on the relationships between performance status, treatment regimens, and nCRT outcomes.

Other studies have identified additional clinical predictors of pCR. Huang et al. [49] and Hamai et al. [50] mentioned that younger age, higher pretreatment hemoglobin levels, smoking status, and shorter tumor length were significant pCR predictors in ESCC specifically. Meanwhile, Patel et al. [51] demonstrated that the presence of signet ring cell histology in the baseline biopsy was associated with lower rates of pCR and survival in esophageal adenocarcinoma. Recently, prediction models and nomograms for nCRT response have expanded. Schneider et al. [52] created a model using histomorphology tumor regression and nodal stage to predict complete surgical resection rates after nCRT in esophageal cancer. These findings highlight the range of potential clinical predictors for neoadjuvant treatment response and outcomes.

In this study, our pCR rate was approximately 32.7%, lower than the 49% rate for squamous cell carcinoma in the CROSS study. This disparity can be attributed to differences in patient factors. The CROSS study predominantly excluded T4 and N2 stages, while some of our patients had advanced disease. Compared to the CROSS study, the overall patient condition was generally poorer in this present study, with approximately 21.5% of patients having an ECOG performance status score of 2. Haisley et al. demonstrated that approximately 37 patients (26%) achieved a pCR according to the final pathology results. T The cisplatin/5-fluorouracil group had a pCR rate of 33%, while the carboplatin/paclitaxel group had a lower rate of 22% [45]. Huang et al. evaluated the independent predictive clinical factors associated with pCR after nCRT for ESCC, and only Fifty-nine (20.9%) of the 282 patients achieved pCR [49]. The pCR rates can vary among different studies and multiple factors have an effect on the pathological response in esophageal squamous cell carcinoma. It is normal to observe pCR rates ranging between 20 and 40%.

The nomogram developed this study provides robust evidence for predicting neoadjuvant chemoradiotherapy efficacy in future esophageal cancer care. However, not all patients are sensitive to neoadjuvant therapy. Expanding clinical utility of existing drugs for different diseases could improve sensitivity in esophageal cancer patients. This novel research direction saves time and resources while ensuring current medication safety. More importantly, it accelerates development of innovative therapeutic approaches [53]. For esophageal cancer drug repurposing, we can refer to a study utilizing machine learning and deep learning across large cancer datasets to systematically identify repurposing opportunities [54]. By screening suitable drugs for each cancer type, their computational framework enables systematic rediscovery for anti-tumor treatment. In addition, with the accumulation of new clinical data, continued optimization and updating of the model will be crucial. Including new cases can avoid retrospective study limitations. We can also selectively incorporate more parameters like lab tests, treatment variables, and genomics to enhance predictive accuracy.

Compared to previous studies, this study has several advantages: first, the analyzed CT images were acquired from the same machine using unified scanning parameters, preventing interference caused by variations in imaging parameters. Second, fivefold cross-validation was employed to more accurately estimate the prediction model. Finally, we minimized inter-rater variation through two radiologists’ independent evaluation, only a few studies examined the reproductivity, reproducibility and reliability errors in radiomics [55, 56].

While the longitudinal images in delta-radiomics demonstrate significant predictive potential, it's important to acknowledge some limitations in this study. First, it is a retrospective study, there exists a bias in the selection and control of patients. Future prospective studies with larger sample sizes are needed to ensure generalization. Second, training and validation datasets were acquired from a single institution, further investigation will concentrate on samples from various institutions as external validation dataset. Additionally, genomics data should be incorporated to associate with treatment response in the future, which might improve the accuracy of this model. Finally, a key limitation of radiomics studies is the lack of clear biological interpretability for radiomic features and their changes, as underlying mechanisms remain unclear. In summary, this study demonstrates the potential impact of a predictive model using radiomics and clinical factors on treatment decision-making for resectable locally advanced ESCC. Further optimization and validation are warranted to translate these findings into clinical practice. In addition, the method of patient grouping still needs to be discussed. Previous studies have shown that randomly splitting patients into training and validation sets with a ratio of 8:2 or 7:3 when building clinical prediction models may lead to unstable model performance [57]. Employing k-fold cross-validation on the sample may be one way to address this issue. Moreover, it can also provide more stable model evaluation and avoid overfitting and underfitting [58]. However, in machine learning models based on radiomics, k-fold cross-validation risks data leakage and introduces errors. Also, inappropriate k values, imbalanced training and validation set division, and unshuffled data order affect model prediction [59, 60]. This study adopted random grouping and k-fold cross-validation for parameter selection during model building. We also introduced model validation, normalization, and preprocessing to ensure model stability. Radiomics research standard protocols and guidelines have not specified criteria for grouping, necessitating further research [61, 62]. But avoiding underfitting and overfitting, improving generalization and interpretability, are key future research goals.

## Conclusions

In conclusion, this preliminary study demonstrated that combining clinical factors and CT delta-radiomics before and after treatment can accurately predict pCR in patients with ESCC receiving nCRT. The nomogram model provides an economical, non-invasive approach to predict neoadjuvant chemoradiotherapy response, which is helpful for guiding clinical treatment decisions. This study suggests radiomic analysis may be a useful tool for individualized assessment of tumor response and personalized therapy selection in locally advanced esophageal cancer.

## Data Availability

The datasets generated or analyzed during the study are available from the corresponding author on reasonable request.

## Abbreviations

CT: Computed tomography
pCR: Pathological complete response
ESCC: Esophageal squamous cell carcinoma
nCRT: Neoadjuvant chemoradiotherapy
ROIs: Regions of interest
LASSO: Least absolute shrinkage and selection operator
DRF: Delta-radiomics features
ROC: Receiver operating characteristic
AUC: Area under curve
DRS: The delta-radiomics signatures
EC: Esophageal cancer
SCC: Squamous cell carcinoma
AI: Artificial intelligence
ML: Machine learning
MRI: Magnetic resonance imaging
PET: Positron emission tomography
AJCC: American joint committee on cancer
IMRT: Intensity-modulated radiotherapy
TRG: Tumor regression grade
IH: Intensity histogram
GLCM: Grey-level co-occurrence matrix
GLRLM: Gray-level run-length matrix
GLSZM: Gray-level size zone matrix
NGTDM: Neighboring gray-tone difference matrix
GLDM: Gray-level dependence matrix
ICCs: Interclass correlation coefficients
PRC: Precision-recall curve
AP: Average precision
DCA: Decision curve analysis
DECT: Dual energy CT
DWI-MRI: Diffusion-weighted imaging magnetic resonance imaging
FDG PET: Fluorodeoxyglucose-positron emission tomography
